# Effects of grazing on spatiotemporal variations in community structure and ecosystem function on the grasslands of Inner Mongolia, China

**DOI:** 10.1038/s41598-017-00105-y

**Published:** 2017-02-28

**Authors:** Rina Su, Junhui Cheng, Dima Chen, Yongfei Bai, Hua Jin, Lumengqiqige Chao, Zhijun Wang, Junqing Li

**Affiliations:** 1 Key Laboratory for Silviculture and Conservation of Ministry of Education, Forest College, Beijing Forestry University, Beijing, 100083 China; 2 College of Grassland and Environmental Science, Xinjiang Agricultural University, Urumqi, 830052 China; 3 State Key Laboratory of Vegetation and Environmental Change, Institute of Botany, Chinese Academy of Sciences, Beijing, 100093 China; 4 Institute of Grassland Surveying and Planning, Inner Mongolia, Hohhot 010051 China

## Abstract

Grasslands worldwide are suffering from overgrazing, which greatly alters plant community structure and ecosystem functioning. However, the general effects of grazing on community structure and ecosystem function at spatial and temporal scales has rarely been examined synchronously in the same grassland. Here, during 2011–2013, we investigated community structure (cover, height, and species richness) and aboveground biomass (AGB) using 250 paired field sites (grazed *vs*. fenced) across three vegetation types (meadow, typical, and desert steppes) on the Inner Mongolian Plateau. Grazing, vegetation type, and year all had significant effects on cover, height, species richness, and AGB, although the primary factor influencing variations in these variables was vegetation type. Spatially, grazing significantly reduced the measured variables in meadow and typical steppes, whereas no changes were observed in desert steppe. Temporally, both linear and quadratic relationships were detected between growing season precipitation and cover, height, richness, or AGB, although specific relationships varied among observation years and grazing treatments. In each vegetation type, the observed community properties were significantly correlated with each other, and the shape of the relationship was unaffected by grazing treatment. These findings indicate that vegetation type is the most important factor to be considered in grazing management for this semi-arid grassland.

## Introduction

Overgrazing has led to extreme changes in the community structure and ecosystem functioning of many grasslands^1–3^. On a global scale, researchers have estimated that 60% of grasslands suffer from grazing or overgrazing, and that these conditions are more serious in some areas with high populations of grazing animals^4^. For example, as the largest ecosystem type in China, grasslands cover 393 million ha, or 42% of the national territory^5^. During the past three decades, in the grassland ecosystems in China, unsustainable land management techniques, such as overgrazing, have caused serious desertification and salinization^6^. For example, in the grasslands of Inner Mongolia, which has the largest area of grassland in China, approximately half of the grassland area has suffered from grazing-induced degradation during the past three decades^7^. Previous studies have independently demonstrated that grazing alters the community structure and ecosystem function of grasslands both at spatial and temporal scales, but the effects of grazing vary among grassland types and observed years^4, 8–11^. These results have indicated that grazing-induced variations in community structure and function are more complex than originally thought and depend on both grassland context and changes in climatic conditions among observation years. Thus, it is anticipated that simultaneously studying and comparing grazing-induced variation in community structure and function at both spatial and temporal scales in a given grassland will further improve our understanding for mitigation and adaptation strategies in response to climate change in this field.

A number of studies have demonstrated that, at different spatial scales or vegetation types, grazing significantly altered community structure (i.e., vegetation cover, community height, and species richness) and ecosystem functioning (aboveground biomass: AGB) in grasslands^12, 13^. At different spatial scales, the patterns and magnitude of the changes in cover, height, species richness, and AGB in response to grazing have been shown to be mediated by vegetation type and climatic conditions^14–16^. Researchers have generally believed that grazing will reduce cover, height, and AGB in most vegetation types^17^, whereas species richness will only decrease under benign conditions (i.e., meadow steppe and typical steppe) and grazing will have no significant effects on species richness under harsh conditions (i.e., desert steppe and desert^18^). Recent reports have also demonstrated that these responses varied depending on different locations with different climatic conditions in grassland ecosystems^19, 20^. For example, the response of ANPP to gazing at dry sites are more vulnerable to wet sites, particularly in semi-arid grasslands^20^. These results indicate that the spatial response of community structure and ecosystem function to grazing are context dependent, and may be influenced by factors such as elevation and the nutrient content, moisture, and bulk density of soil. In addition to vegetation type, the responses of cover, height, species richness, and AGB at various spatial scales have also been shown to be influenced by climatic gradients. For example, with increasing precipitation, the trends in species richness and AGB in the Inner Mongolian grasslands have been reported to have changed from a linear form in fenced sites to unimodal relationships in grazed sites^21^. These observations indicate that climatic conditions also play an important role in regulating the responses of community structure and ecosystem function to grazing. By considering the combined findings of these studies, it is further suggested that both biotic (i.e., vegetation type) and abiotic factors (i.e., climatic gradients) should be explored when explaining the influence of grazing on community structure and ecosystem function.

In addition to the spatial scale, temporal scale adds another important dimension to understand the effects of grazing on community structure and ecosystem function. For example, independent meta-analyses showed that community cover and AGB responded negatively to grazing, but that plant species richness responded neutrally at a temporal scale^10, 22, 23^. In addition to the differential responses of these variables to grazing, they are also commonly modified by interannual variability in climatic conditions, such as rainfall, aridity, and microenvironmental variables^10, 11, 22, 23^. These results indicate that the responses of community structure and function to grazing are considerably more complex than originally thought, and that they are controlled by multiple factors, including grazing conditions, vegetation type, and climatic conditions. However, we still do not know which among these factors is the most important in terms of regulating the responses of community structure and function to grazing, and thus additional studies are needed.

During the past decade, China has implemented a series of national project-related strategies designed to control grassland degradation and to improve grassland conditions. One important strategy is a nationwide conservation project known as Returning Grazing Lands to Grasslands (RGLG). RGLG aims to restore these damaged ecosystems to a healthy state and promote an equilibrium between ecological protection and socioeconomic development^24, 25^. The RGLG program has established a large number of paired sites (grazed *vs.* fenced plot) in national grasslands to continuously monitor the recovery of cover, species richness, community height, and AGB. These paired sites include most of China’s grassland types and cover a wide range of climatic conditions, thereby providing an ideal system in which to compare the spatial and temporal responses of community structure and ecosystem function in grasslands.

**Figure 1:**
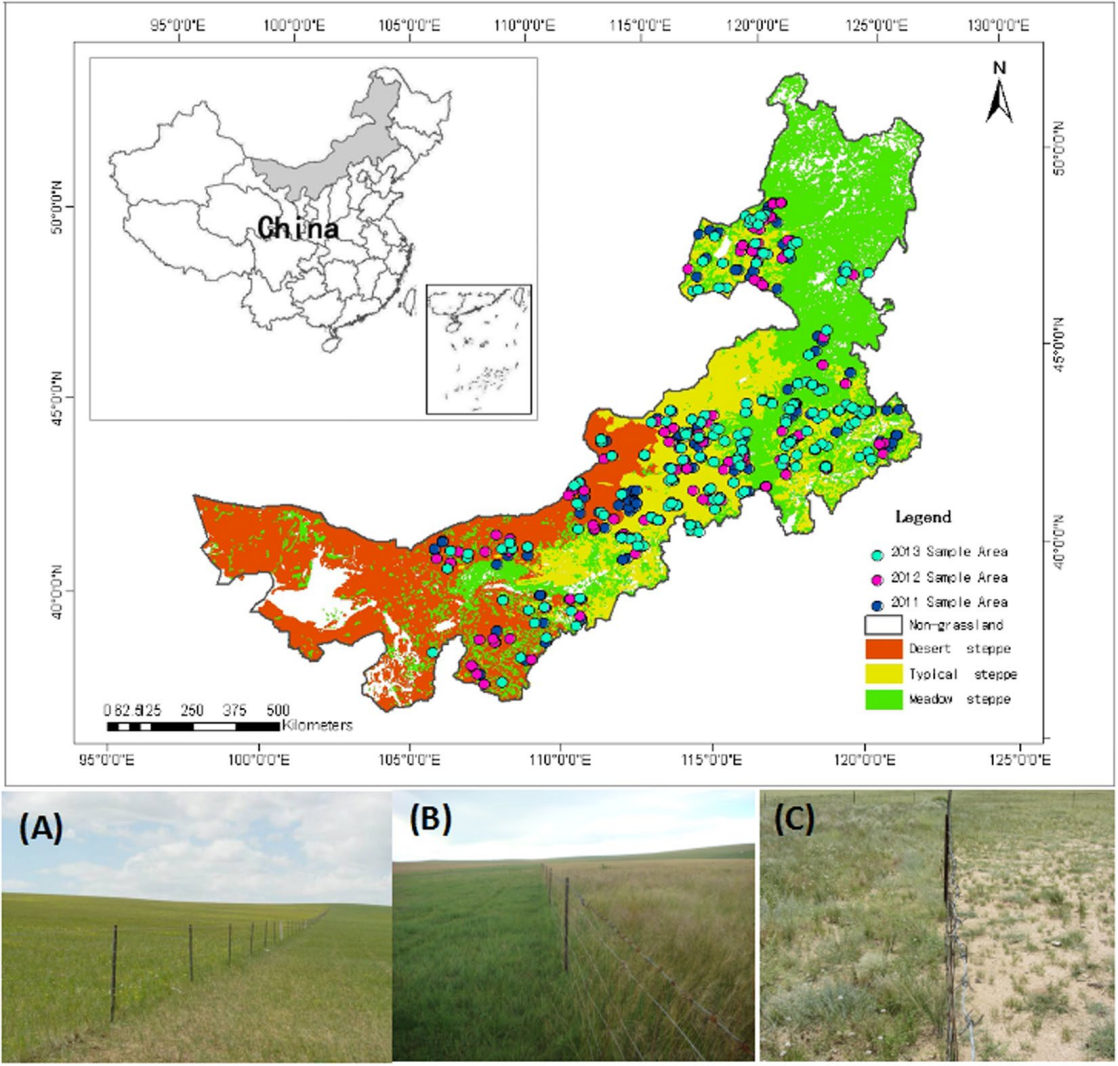
Locations of the 250 paired (fenced and grazed pairs) study sites in Inner Mongolia. (**A**), Meadow steppe (**B**), typical steppe (**C**), desert steppe. Map created using ArcGIS 9.3 software. (Environmental Systems Research Institute (ESRI), Redlands, CA, USA).

Using a long-term dataset from 250 paired sites (grazed *vs.* fenced) in three grassland types (meadow steppe, typical steppe, and desert steppe) in Inner Mongolia (Fig. 1), we investigated changes in the spatial and temporal patterns of community structure and ecosystem function in response to grazing and precipitation. Specifically, we addressed the following three questions: (1) How does grazing affect community structure (species richness, height, and cover) and function (aboveground biomass: AGB) among vegetation types (spatial scale) and among years (temporal scale) in the Inner Mongolian grasslands? (2) How does grazing affect the forms of the relationships between community properties and precipitation in the observation years? (3) How does grazing affect the forms of the relationships among community properties in different vegetation types?

## Results

### Effects of grazing, vegetation type, and year on community properties

The results of three-way ANOVA showed that community cover, height, species richness, and AGB were significantly affected by the main effects of grazing treatment, vegetation type, and year, with the exception of the effect of year on species richness (all *P* < 0.05, Table 1). However, variation in these variables was mainly explained by vegetation type (12.38–38.31%) and grazing treatment (4.49–13.22%), whereas year explained only 0.28–4.17% of the variation (Table 1).

**Table 1:** Results of three-way ANOVAs using grazing treatment, vegetation types and years as fixed factors.

Sources	df	Cover (%)		Height (cm)		Species richness		AGB (g m^−2^)	
		*P*	SS%	*P*	SS%	*P*	SS%	*P*	SS%
Grazing (G)	1	**<0.0001**	6.70	**<0.0001**	11.30	**<0.0001**	3.49	**<0.0001**	13.22
Vegetation types (V)	2	**<0.0001**	38.31	**<0.0001**	14.38	**<0.0001**	12.38	**<0.0001**	18.34
Years	2	**0.0211**	0.28	**<0.0001**	4.17	0.0835	0.27	**<0.0001**	1.04
G*V	2	**0.0032**	0.42	**<0.0001**	1.42	**0.0195**	0.44	**<0.0001**	1.66
G*Y	2	0.5589	0.04	0.357	0.09	0.7043	0.04	0.433	0.07
V*Y	2	**0.0266**	0.40	**0.0213**	0.53	**0.0004**	1.16	0.233	0.25
G*V*Y	4	0.8661	0.05	0.8264	0.07	0.6407	0.14	0.882	0.05

df, P, SS% were the abbreviations of degree of freedom, P values and percentage of sum square explained by sources, respectively.

**Figure 2:**
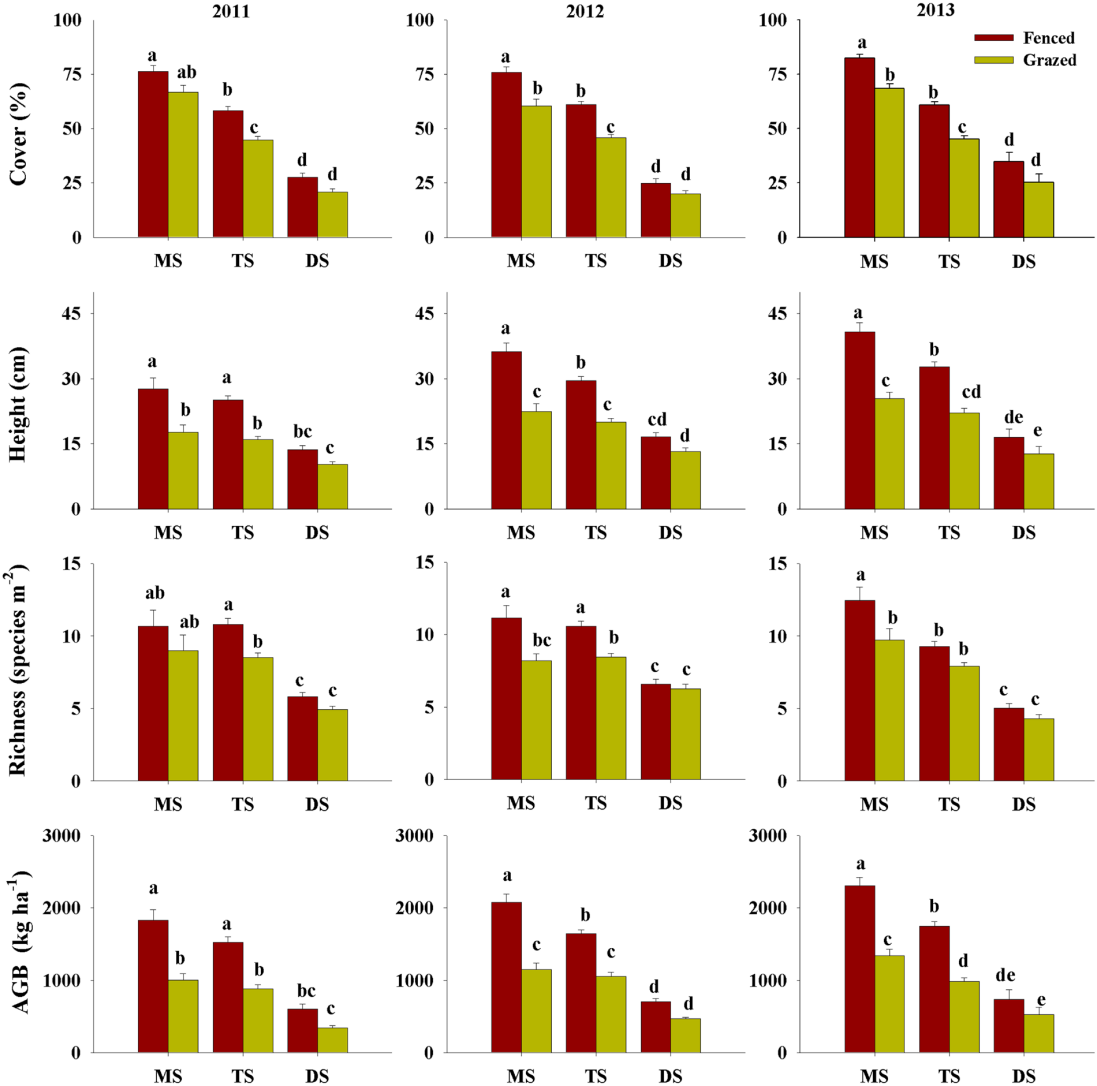
Variation in vegetation cover, height, richness and aboveground biomass (AGB) among three vegetation types (MS: meadow steppe; TS: typical steppe; DS: desert steppe) in 2011–2013.

Compared with the fenced plots, community cover, height, species richness, and AGB declined considerably in the grazed plots of meadow and typical steppe during the period 2011–2013, with the exception of cover and species richness in meadow steppe in 2011 and species richness in typical steppe in 2013 (Fig. 2). On average, community cover, height, species richness, and AGB in grazed plots decreased by 17 ± 1% (mean ± standard error), 37 ± 2%, 21 ± 2%, and 44 ± 2%, respectively, in meadow steppe, and by 41 ± 1%, 26 ± 2%, 18 ± 1%, and 41 ± 2%, respectively, in typical steppe, during the period from 2011 to 2013.

In addition to the influence of the main effects, community cover, height, species richness, and AGB were also significantly affected by the interactive effects of grazing treatment × year and vegetation type × year, with the exception of interactive effects of vegetation type × year on AGB (all *P* < 0.05, Table 1), demonstrating that the effects of grazing treatment on community properties and function depend on vegetation type. This was further affirmed by the fact that the grazing-induced decrease in cover, height, species richness, and AGB in meadow steppe was different to that in typical steppe, as mentioned above.

**Figure 3:**
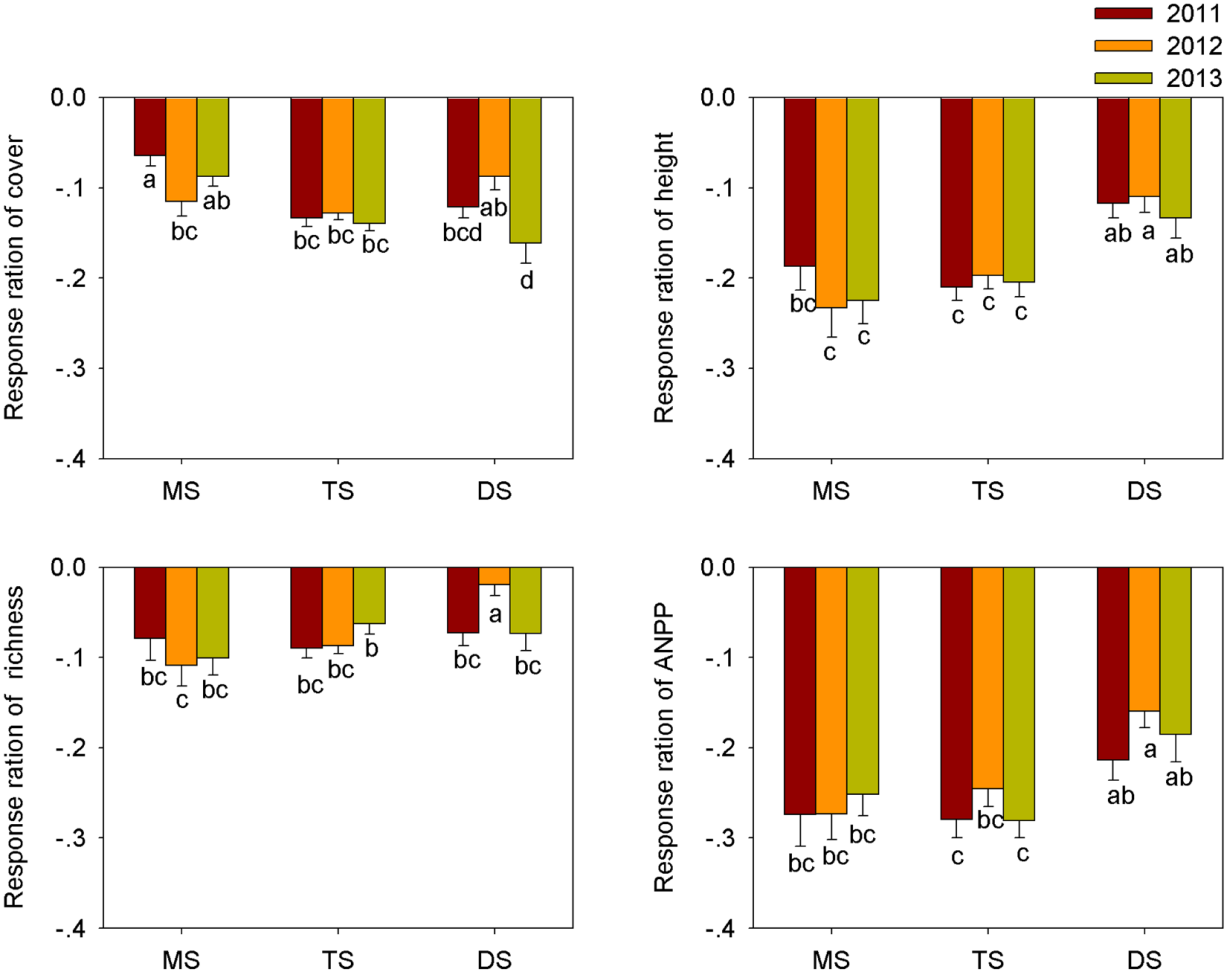
Spatial pattern of grazing-induced response in vegetation cover, height, species richness and AGB among three vegetation types (MS: meadow steppe; TS: typical steppe; DS: desert steppe) in 2011–2013.

To further explore the temporal variation in grazing-induced variation among vegetation types, we compared the response ratios [RRs, indicated by ln (C_G_/C_F_), where C_F_ and C_G_ are the mean values of the community properties in fenced and grazing plots at each site, respectively] of cover, height, species richness, and AGB over the period 2011–2013 for each of the vegetation types (Fig. 3). Our results showed that for a given vegetation type, there was no significant variation in the RRs of cover, height, species richness, and AGB during the years 2011 to 2013, with the exception that meadow steppe had the lowest RRs of cover in 2012 and desert steppe had the highest RRs of species richness in 2012 (Fig. 3). Moreover, the RRs of species richness and AGB of meadow steppe and typical steppe were significantly lower than those of desert steppe during the period 2011–2013 (Fig. 3).

### Effect of grazing on the relationships between community properties and precipitation

**Figure 4:**
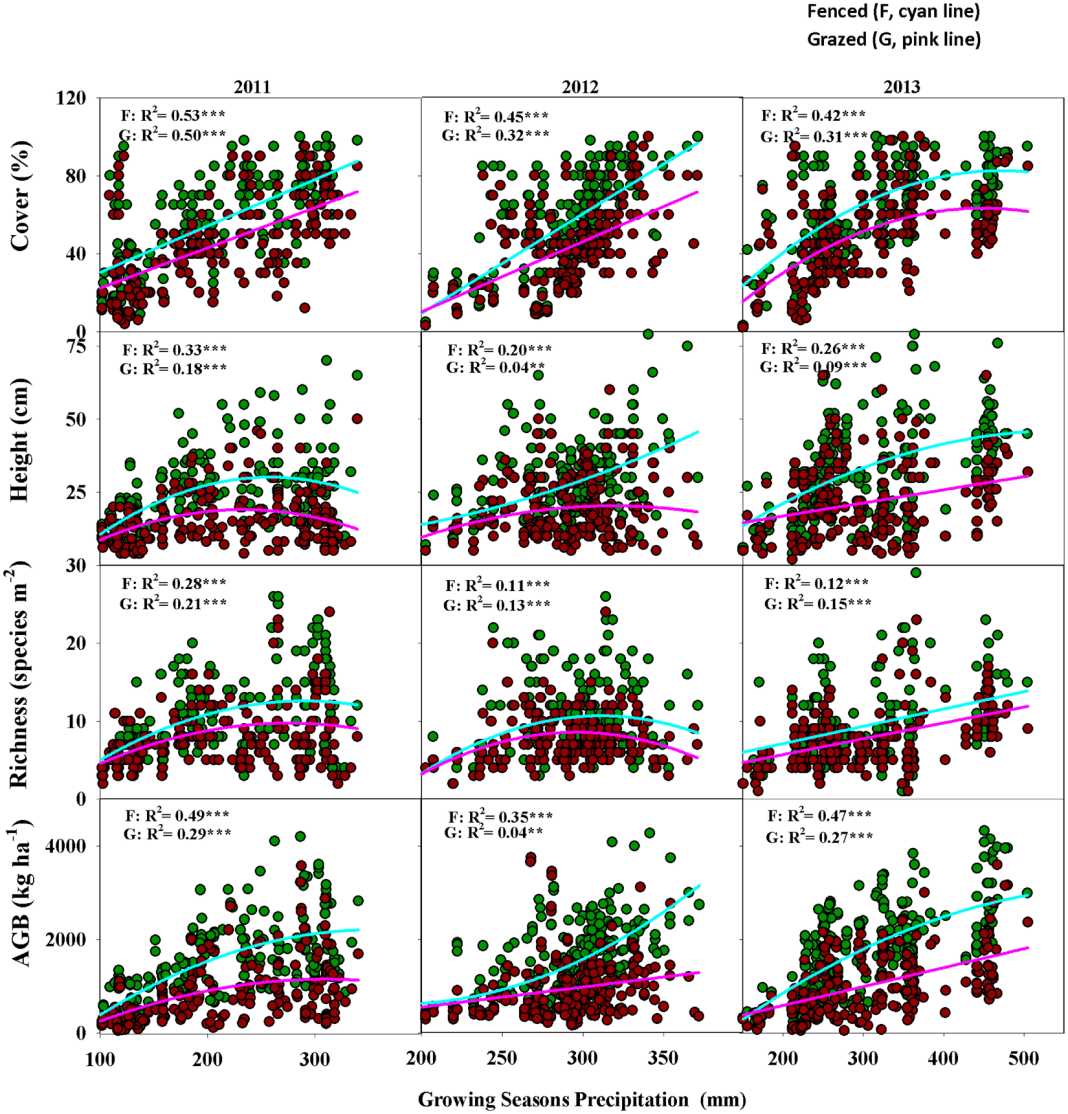
Relationship between vegetation cover, height, richness, aboveground biomass (AGB) and growing season precipitation at both fenced (**F**) and grazed (**G**) sites. *P-value < 0.05, **P-value < 0.01, ***P-value < 0.0001. MS: meadow steppe; TS: typical steppe; DS: desert steppe.

Our results showed that the forms of the relationships between community properties and precipitation were influenced by grazing treatment and by the year of observation (Fig. 4). Irrespective of grazing treatment, the relationships between community cover and growing season precipitation (GSP) changed from a positive linear form in 2011 and 2012 (relatively low GSP, 350 and 400 mm, respectively) to a unimodal form in 2013 (relatively high GSP, 500 mm, Fig. 4). In contrast, the relationship between species richness and GSP changed from a unimodal form in 2011 and 2012 to a positive linear form in 2013 (Fig. 4). Furthermore, community height and AGB consistently exhibited unimodal relationships with GSP in fenced plots during the period 2011–2013 (Fig. 4). However, the relationship between species richness and GSP in grazed plots shifted from a unimodal form in 2011 and 2012 to a positive linear form in 2013 (Fig. 4), whereas the AGB in grazed plots also changed from a unimodal form in 2011 to a linear form in 2012 and 2013 (Fig. 4).

### Effect of grazing on the relationships among community properties

**Figure 5:**
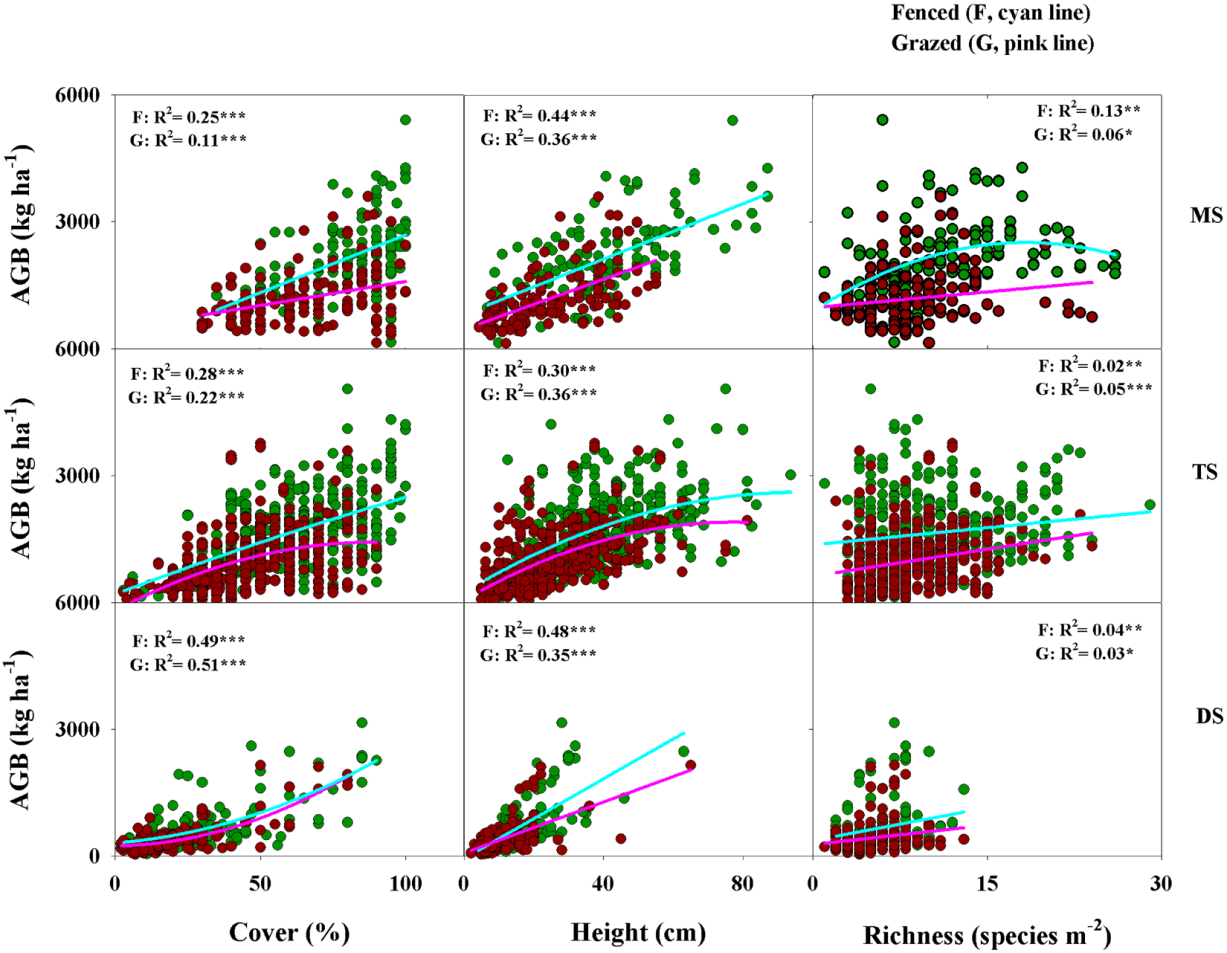
Relationships between aboveground biomass (AGB) and vegetation cover, height, richness at both fenced (**F**) and grazed (**G**) sites. *P-value < 0.05, **P-value < 0.01, ***P-value < 0.0001. MS: meadow steppe; TS: typical steppe; DS: desert steppe.

In addition to the significant relationship between community properties and GSP, community properties were also found to be correlated with each other in each vegetation type. However, relative to the fenced plots, grazing did not alter the forms of the relationships between AGB and cover, height, and species richness (Fig. 5). For example, a positive linear relationship was consistently found between AGB and cover, height, and species richness in both fenced and grazed plots (Fig. 5), with the exception that a unimodal relationship was found between AGB and species richness in fenced plots in meadow steppe, between AGB and height in typical steppe, and between AGB and cover in desert steppe (Fig. 5).

## Discussion

### Effects of grazing, vegetation type, and year on community properties

It has been well demonstrated that high grazing intensity could trigger a significant and destructive effect on community composition and ecosystem functioning in grasslands, such as decreases in community cover and AGB^26, 27^, which is consistent with our findings for Inner Mongolian grassland. However, some previous studies have indicated that the relationships between plant variables (e.g., biomass or productivity) and grazing intensity are hump-shaped in grassland ecosystems^19, 20^. For example, Dangal *et al*.^20^ provided evidence that grassland ANPP can be maintained at a grazing intensity of 0.5 and 1.0 sheep ha^−1^ at wet and dry sites in in semi-arid regions in Central Asia, respectively. The absence of grazing improving plant variables in the current study may be due to the relative higher grazing intensity (*ca.* 2.0 sheep ha^−1^) in Inner Mongolian grasslands. Hence, the patterns observed in our semiarid grasslands may simply represent the right side of a hump-shaped relationship. Moreover, our results also showed that in addition to grazing, community cover, height, species richness, and AGB were also significantly influenced by vegetation type and the interactive effect of grazing × vegetation type, indicating that at a spatial scale, grazing-induced decreases in cover, height, species richness, and AGB were context dependent. We found that grazing significantly decreased cover, height, species richness, and AGB in meadow and typical steppe, but had no significant effect in desert steppe, which is consistent with the findings of some previous studies in this region, which have reported that the effects of grazing on community properties are strongly mediated by vegetation type^21, 24, 28^. These different response patterns could be explained by variation in community composition among the vegetation types and the foraging performance of livestock. In particular, the meadow steppe and typical steppe investigated in our study are dominated by species such as *C. pediformis, L. chinensis*, and *S. baicalensis* that are palatable to most livestock, whereas desert steppe is dominated by species such as *S. glareosa* and *S. klementzii* that are unpalatable to livestock. These two factors in turn lead to grazing-induced decreases in cover, height, species richness, and AGB in meadow steppe and typical steppe that are greater than those in desert steppe. Inconsistent with our findings, however, are the results from previous studies that have shown that meadow steppe was less affected by grazing than desert steppe^29, 30^. These discrepancies serve to indicate the complexity of the responses of community properties to grazing, which are controlled by multiple factors in addition to grazing intensity, including grazing history, variation in climatic conditions during the years of observation, and soil properties^31–33^.

Our results also revealed that, with the exception of species richness, community properties changed among the years of observation, which is consistent with the findings of a previous study in Mongolian grassland neighboring our study area^34^. This similarity is because species in the grasslands of the Mongolian plateau are dominated by perennials, which have relatively slow temporal dynamics^34^. Moreover, we found significant interactive effects between years and vegetation types, indicating that the response of community properties to grazing at the temporal scale was also mediated by vegetation context. This conclusion is also affirmed by the variance component results, which revealed that vegetation type, rather than grazing treatment or year, was the most important factor in regulating the variation in community properties during the period in which we studied the Inner Mongolian grassland. This finding is consistent with the previous studies^19, 20, 35^, which predict that the plants at dry sites are deferent to wet sites because grazing would change precipitation use efficiency and nitrogen use efficiency. Our results not only expand our understanding of the importance of community responses to grazing but also have important implications for grazing management in the context of different vegetation conditions in the semiarid steppe and beyond. For example, we should decrease the grazing intensity for conservation of biological diversity in the Inner Mongolian grasslands.

### Effect of grazing on the relationships between community properties and precipitation

Relationships between community properties and climatic gradients have long been debated in ecology. The reported type of relationship has varied from unimodal^36, 37^ to monotonous (positive or negative linear)^38–40^ and to unrelated patterns^41^. However, our results from the Inner Mongolian grasslands revealed the existence of both monotonous and unimodal forms of relationships between cover, height, species richness, and AGB and GSP, although the specific form of the relationship varied among years and grazing treatments. Specifically, we found that the relationship between cover and GSP shifted from positively linear in the dry years (2011 and 2012) to unimodal in the wet year (2013), irrespective of grazing treatment, whereas the relationship between species richness and GSP changed from unimodal in the dry years to linear in the wet year, indicating that the form of the relationships between cover and species richness and climatic gradient is mediated by the temporal variation in climatic conditions. We noted that this observed difference in relationship between species richness and GSP in our study is inconsistent with the findings of some previous studies in this area^42–44^. This discrepancy could be explained by differences in the method of precipitation data collection. Previous authors have only investigated richness in a single given year and used mean annual precipitation to explore their relationships. In our study, we surveyed species richness during three consecutive years (two relatively dry years followed by one relatively wet year) and obtained GSP data for each site in each year. This method allowed us to detect temporal variations in the relationship between species richness and GSP, because species richness changed differently in the wet year after the grasslands had experienced some dry years^45^.

We also noted that the forms of the relationships between height and AGB and GSP were affected by grazing treatment. Grazing changed these relationships from a unimodal to linear form in some observation years (i.e., for height in 2013 and for AGB in 2012 and 2013). This finding is consistent with a previous study in this region, which reported that grazing could alter the relationship between AGB and precipitation^21^. Although we have yet to clarify the underlying mechanisms involved in regulating the forms of these relationships, our results strongly demonstrated that in Inner Mongolian grassland, the form of the relationships between cover, height, species richness, and AGB and GSP are mediated by both grazing and the temporal variation in GSP.

### Effect of grazing on the relationships among community properties

In this study, we found that AGB exhibited significant relationships with cover, height, and species richness, irrespective of vegetation type. Our findings indicate that some of the previous seemingly inconsistent observations in the Inner Mongolian grassland are in fact not conflicting, but instead reflect synchronous relationships. For example, we identified a unimodal relationship between AGB and species richness in meadow steppe, whereas a linear relationship was detected in typical steppe and desert steppe. These findings differ from those of some other studies, which have reported that the relationship between AGB and species richness is invariably of a unimodal or linear form^46, 47^. Our results are, however, in line with an early hypothesis, which proposed that the forms of relationships between AGB and species richness vary among vegetation types due to variations in community composition and environmental conditions^48^. Moreover, we also found that, compared with the fenced plots, grazing generally did not affect the relationships between AGB and cover, height, and species richness. This could be explained by the fact that grazing decreased all investigated variables in each grassland type, and thus did not influence the intrinsic relationship among these community properties.

In this study, we performed a large-scale experiment using 250 paired plots (grazed *vs.* fenced) to survey community properties (cover, height, species richness, and AGB) across three vegetation types (meadow steppe, typical steppe, and desert steppe) in Inner Mongolian grassland. Our goal was to evaluate the spatial and temporal effects of grazing on these variables in this region. Our results showed that in addition to grazing, vegetation type and year also had significant effects on cover, height, species richness, and AGB, and that the primary factor influencing variation in these variables was vegetation type. Moreover, the relationships between community properties and GSP were regulated by both grazing treatment and the year of observation. In each vegetation type, the observed community properties were significantly correlated with each other, and the form of the relationships was unaffected by grazing treatment. Our findings indicate that vegetation type plays an important role in regulating the response of community properties to grazing in this semi-arid grassland.

## Materials and Methods

### Study area and experimental design

This study was conducted in the grasslands of Inner Mongolia, which are an important part of the Eurasian steppe region (37.4°–53.38°N, 97.2°–126.07°E, Fig. 1), covering an area of 1.18 million km^2^ ^49^. The grasslands of Inner Mongolian experience a continental monsoon climate, with an uneven rainfall pattern and a hot but relatively short frost-free period. From the western to eastern regions, the mean annual precipitation (MAP) in these grasslands gradually increases from 50 to 450 mm, with 70–80% of the MAP occurring during the growing season (May–August). Moreover, the mean annual temperature gradually decreases from 8 to 0 °C. With a variation in climatic conditions, soil types correspondingly change (west to east) from desert soils to brown calcic to chernozem soils.

Our study included three dominant vegetation types growing in the Inner Mongolian grasslands: meadow steppe, typical steppe, and desert steppe. The meadow steppe is located in the eastern part of the Inner Mongolian grasslands and is characterized by a relatively high species richness and productivity. Typical species include *Carex pediformis* C. A. Mey.*, Filifolium sibiricum* (L.) Kitam*., Leymus chinensis* (Trin.) Tzvel., *Sanguisorba officinalis* L, and *Stipa baicalensis* Roshev. The typical steppe is located in the center of the Inner Mongolian grasslands and is characterized by an intermediate level of species richness and productivity. Typical species include *Artemisia frigida* Willd. Sp. P., *Cleistogenes squarrosa* (Trin.) Keng., *L. chinensis*, *Stipa grandis* P. Smirn., *Stipa krylovii* Roshev, and *Thymus mongolicus* Ronn. The desert steppe is distributed in the western part of the Inner Mongolian grassland and is characterized by relatively low species richness and productivity. Typical species include *Allium polyrhizum* Turcz. ex Regel, *Stipa caucasica* Schmalh, and *Stipa klemenzii* Roshev^21, 50, 51^.

### Experimental design and field measurement

On the basis of the spatial distribution of vegetation types and community associations, the RGLG project established a total of 250 paired sites (grazed *vs.* fenced) in the Inner Mongolian grassland, including 63 paired sites for meadow steppe, 148 paired sites for typical steppe, and 39 paired sites for desert steppe (Fig. 1). Each of these paired sites included two neighboring but contrasting plots: one fenced and one grazed. Since 2003, all the fenced plots have been enclosed by wire netting to prevent livestock grazing, whereas grazed plots are open and have suffered from overgrazing for a long period.

For the years 2011 to 2013, we investigated community structure (cover, height, and species richness) and function (aboveground biomass, AGB) in these 250 paired sites during the period from August 15^th^ to 30^th^ when the aboveground standing biomass of most plants peaks in this region^43, 51^. For each paired site, we initially recorded its longitude, latitude, elevation, and community type. To minimize the spatial heterogeneity, we randomly selected three 1 × 1 m quadrats in each of the fenced and grazed plots at each site to investigate their community cover, height, species richness, and AGB. Then, we pooled the data from the three quadrats at site level for the following statistical analyses. Specifically, community cover was assessed by a visual inspection, average community height for each quadrat was measured using a portable ruler, and species richness was determined by counting the number of species present in the quadrats. After measuring these variables, we clipped the AGB at ground level in each quadrat, oven-dried the vegetation at 65 °C for 48 h, and then weighed the dried material in the laboratory.

### Climatic data

We used growing season precipitation (GSP) to reflect climatic conditions in our study, because previous studies have affirmed that it is the primary climatic factor influencing community structure and ecosystem function in this area^43, 51^. To obtain the GSP for these paired sites, we initially collected monthly precipitation data for the years 2011 to 2013 from 824 evenly distributed meteorological stations in China. On the basis of these collected data, we then interpreted monthly precipitation data for our investigated site via a Geographic Information System-based multiple regression method, which used latitude, longitude, and elevation as predicators^52^. Finally, the GSP at each site was calculated by summing the interpreted monthly precipitation data from May to September.

### Statistical analyses

To address our first question regarding the influence of grazing, vegetation type, and year on community structure and ecosystem function, we used a three-way ANOVA to examine the main and interactive effects of grazing, vegetation type, and year on variations in cover, height, species richness, and AGB at the plot level, respectively. If a significant main or interactive effect was detected, we then calculated its relative importance by dividing its explained sum square by the total sum square^53^, and then Tukey’s multiple-range test was used to compare differences in the mean values among these factors. In addition, to assess grazing-induced variation in community structure and ecosystem function, we also compared the differences in response ratios of community cover, height, species richness, and AGB among different vegetation types in each year of observation. The response ratio was calculated as RR = ln (C_G_/C_F_), where C_F_ and C_G_ are the mean values of the community properties that were observed in fenced and grazing plots at each site, respectively.

To address our second question regarding the effect of grazing on the forms of the relationships between community properties and GSP for each community property (i.e., cover, height, species richness, and AGB), we initially used a general linear model to examine whether a significantly positive (linear term) or unimodal (quadratic term) relationship existed. If the quadratic term was not statistically significant, we then considered that a linear relationship was the best-fitted model between community properties and GSP. If a significant quadratic term was also detected, we then used the Akaike Information Criterion (AIC) value to select the best-fitted model (model with the lowest AIC)^46^. Similarly, we also used this procedure to address our third question regarding the effect of grazing on the relationships among community properties. All statistical analyses were performed with R version 2.15.1 (R Development Core Team, 2009).
